# SNP Detection in mRNA in Living Cells Using Allele Specific FRET Probes

**DOI:** 10.1371/journal.pone.0072389

**Published:** 2013-09-09

**Authors:** Liya Dahan, Lingyan Huang, Ranit Kedmi, Mark A. Behlke, Dan Peer

**Affiliations:** 1 Department of Cell Research and Immunology, Tel Aviv University, Tel Aviv, Israel; 2 Center for Nanoscience and Nanotechnology, Tel Aviv University, Tel Aviv, Israel; 3 Integrated DNA Technologies, Coralville, Iowa, United States of America; University of North Carolina at Charlotte, United States of America

## Abstract

Live mRNA detection allows real time monitoring of specific transcripts and genetic alterations. The main challenge of live genetic detection is overcoming the high background generated by unbound probes and reaching high level of specificity with minimal off target effects. The use of Fluorescence Resonance Energy Transfer (FRET) probes allows differentiation between bound and unbound probes thus decreasing background. Probe specificity can be optimized by adjusting the length and through use of chemical modifications that alter binding affinity. Herein, we report the use of two oligonucleotide FRET probe system to detect a single nucleotide polymorphism (SNP) in murine *Hras* mRNA, which is associated with malignant transformations. The FRET oligonucleotides were modified with phosphorothioate (PS) bonds, 2′OMe RNA and LNA residues to enhance nuclease stability and improve SNP discrimination. Our results show that a point mutation in Hras can be detected in endogenous RNA of living cells. As determined by an Acceptor Photobleaching method, FRET levels were higher in cells transfected with perfect match FRET probes whereas a single mismatch showed decreased FRET signal. This approach promotes *in vivo* molecular imaging methods and could further be applied in cancer diagnosis and theranostic strategies.

## Introduction

Much effort has been devoted in the past decade to developing nanostructured molecular probes for RNA live cell imaging [1]. In the field of cancer diagnostics and targeted delivery, most research today is aimed at finding extracellular protein markers (Tumor Associated Antigens, TAA) exclusively or differently expressed in tumors. Discovery of new TAAs is extremely challenging and time consuming whereas tumor genetic alterations (TGA) could be applied simply by genomic sequencing. Designing a practical method for monitoring genetic alterations in living cells could be used as a simple method for detection of transformed cells. The main challenge of live genetic detection is the ability to produce a high signal to background ratio with high specificity since unbound probe cannot be removed by stringent washing, as is normally employed in nucleic acid hybridization based assays.

Methods for detection of mRNA in living cells allow real time detection of specific mRNA transcripts [2] as well as endogenous mRNA alterations up to a single base pair resolution [3]. Live cell imaging not only eliminates the need to handle RNA, but also provides an opportunity to analyze gene expression at the single-cell level without arduous fixation, permeabilization, and washing steps.

The ability to detect, localize, quantify and monitor such molecular alterations *in vivo* may set the gold standard for *in vivo* real-time imaging modality assisting in tumor diagnosis and staging. It could be used for applications as early detection of mutated cells in the pre-cancerous stage [1], residual disease and metastases detection found in sites other than the primary tumor and real time identification of tumor margins left after resection, which is one of the main reasons for cancer relapse [1].

Fluorescently labelled antisense oligonucleotides can bind to natural mRNA in a sequence-specific manner and enable real time monitoring of mRNA transcripts in the cell [4]. mRNA based detection of genetic alterations takes advantage of the high degree of specificity in Watson-Crick based base pairing, which was naturally designed and optimized by evolution,thus in principle, allowing high degree of specificity, even in a single base pair resolution as in the endogenous process of hybridization of RNA primers or microRNAs.

Several classes of labelled oligonucleotides (ODNs) have been developed for RNA detection in living cells, including tagged linear oligonucleotide, dual linear fluorescence resonance energy transfer (FRET) oligos, dual-labelled hairpin oligos (e.g. molecular beacons); dual FRET molecular beacons and oligos that use fluorescent reporter proteins [5].

Various studies indicate the high potency of linear antisense ODNs in visualizing cytoplasmic mRNAs [4]. A fluorescent linear antisense oligo can bind to mRNA in a sequence-specific manner. Due to the lack of intramolecular interaction, this antisence oligo has superior hybridization kinetics that enables the dynamic fluctuation of endogenous mRNAs to be detected. However, in order to detect mRNAs with high specificity, it is required to eliminate the fluorescence of unbound oligos from that emitted by oligos bound to target mRNA. When working with live cells or tissues, it is impossible to remove unbound oligos by washing the cells after transfection, therefore an alternative approach must be used in order to enable detection of the desired signal and enhance signal to noise ratio.

Batish et al. recently described a protocol that enables imaging and localization of endogenous mRNAs in situ using multiple linear antisense probes. The use of multiple probes enabled overcoming the high background produced by the free unbound probes allowing enhancement of the signal to noise ratio [6].

Molecular beacons (MB) are antisense molecules possessing complementary sequences on either end of a ODN, enabling the molecule to assume a hairpin configuration in which a fluorophore and quencher are held in close proximity. Hybridization with target nucleic acid sequence opens the hairpin structure and spatially separates the fluorophore from quencher, allowing a fluorescence signal to be generated upon fluorophore excitation only when the MB and target are bound, therefore lowering unspecific background signal. MBs were shown to allow detection of endogenous cytoplasmic mRNA targets in living cells and show advantageous in imaging RNAs owing to the simplicity and specificity of detection [7]. The MBs stem and loop structure, specifically the stem length, is a form of conformational constraint that imparts the MB its selectivity: the MB targeting sequence, which is found in the loop region, would hybridize to the target only if a significant energy gain is offered [8]. In order to perform mismatch discrimination MB probes with longer stems are required since the MB needs to open up only in case of a perfect match,thus, more energy is invested in its opening. The price of increase in stems length is decrease in hybridization rates. Thus, MBs are not suited for monitoring rapidly changing levels of target mRNA and real time detection of SNPs [3].

**Figure 1:**
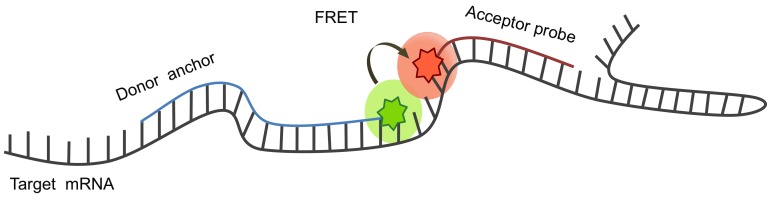
Fluorescence resonance energy transfer probes (FRET) enable the distinction between bound and unbound probes upon hybridization of a pair of linear antisense ODNs to adjacent sequences on target mRNA. When donor anchor is excited and both ODNs are fully complementary to target mRNA, a distance of 4

Another simple method to enhance the signal in an un-washable living system is by the use of fluorescence resonance energy transfer ODNs (FRET); the detection of FRET upon hybridization of a pair of linear antisense oligos to adjacent sequences on a target mRNA (Figure 1) enables the distinction between bound and unbound oligos [9]. Using this technique, detection of cytoplasmic c-fos mRNA utilizing linear antisense oligo deoxynucleotide (ODN) was empowered [2]. Due to the kinetic rate and specificity of linear probes we chose them for the purpose of point mutation detection described herein.

The Hras oncogene appears to be a primary target gene in the formation of Squamous Cell Carcinoma (SCC) in murine skin model and is susceptible to point mutations in several other types of cancers [10]. Hras is a GTPase signaling protein involved in cell division in response to growth factors. Mutation in the second nucleotide of codon 61 causes CAA (gln) to become CTA (Leu) resulting in a constitutively active Hras protein. Hras constant activation causes uncontrolled proliferation of damaged cells and can give rise to tumor formation [11].

We designed a FRET system with two chemically modified linear oligonucleotides to detect a single point mutation in the *Hras* oncogene mRNA in living cells. This system comprises a 14 bp anchor oligo labled with a donor fluorophore, which is complementary to a constant region in the Hras mRNA. The anchor oligo binds both wt (codon CAA) and mutant (CTA) target sequences equally well. Discrimination between wt and mutant targets is achieved by two 8 bp allele-specific probes, labled with an acceptor fluorophore. The probe oligo bind adjacent to the anchor and span the site of the Hras SNP target sequence (Figure 1). Probe A, is fully complementary to the wt (A) target sequence whereas probe T is complementary to the mutant (U) target sequence. The allele specific probe is designed to bind only its perfect match target proximal to the anchor-binding site and as a result exhibit a FRET response.

## Materials and Methods

### Nucleic Acid Probes Design and Synthesis

**Figure 2:**
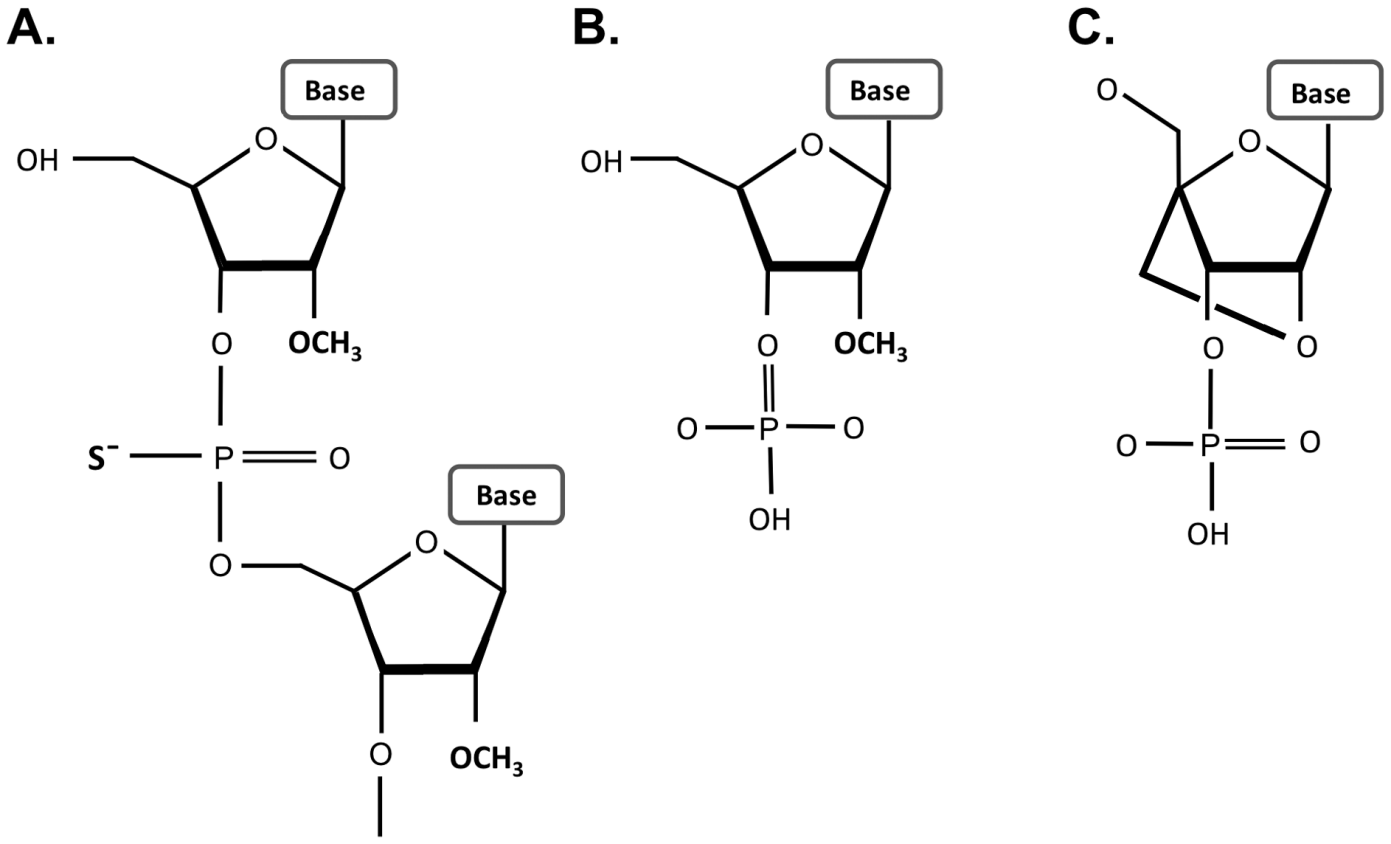
Chemical modifications on the nucleic acid backbone increase their nuclease stability. (**A**) PS bonds include replacement of non-bridging oxygen with sulfur. (**B**) 2′OMe are modification of the 2′-position of the ribose with a methyl group. (**C**) LNA bases contain a methylene bridge between the 2′-*O* with the 4′-C of the ribose and “locks” the sugar conformation.

All ODNs were obtained from Integrated DNA Technologies (Coralville, IA, USA) and were HPLC purified. Probe sequences were selected from murine Hras entries in the Genebank database [NM_008284]. Alexa Fluor 488 dye (AF488) was attached to the anchor oligo on its 3′-end and either Texas Red (TR) or Alexa Fluor 594 (AF594) dyes were attached to the 5′-end of the allele specific probes. The anchor oligo was made of 2′OMe RNA with PS internucleotide linkages. The allele-specific probes were made of 2′OMe RNA with three consecutive Locked Nucleic Acid (LNA) residues positions at the SNP site, as recommend by You et al. [12] (Figure 2). The identity of the oligos were confirmed by electrospray ionization mass spectrometry (ESI-MS). The quality of the oligos were evaluated using analytical HPLC and were all >90% in purity. The quantity of the oligos were determined from UV absorption at 260 nm.

### Cell Lines Growth and Maintenance

308 cells are BALB/c mouse keratinocytes cell line derived from an early stage papilloma of a well-known SCC skin model. This model of multi-stage chemical carcinogenesis represents one of the best-established *in vivo* models for the study of the development of tumors. During the first stage of chemically induced skin carcinogenesis, referred to as ‘initiation’, key genes in epidermal keratinocytes acquire mutations as a result of exposure to a chemical mutagen applied topically, most commonly DMBA (7,12-dimethylbenz [a] anthracene). Activating mutations in *Hras1* can be detected in the epidermis as early as 3–4 weeks after treatment with DMBA and are observed in the majority of papillomas that develop after the second stage called ‘promotion’ which is induced by topical treatment with TPA (12-O-tetradecanoylphorbol-13-acetate) [11]. 308 cells carry the Hras A>T point mutation on the 2^nd^ nucleotide of codon 61 in one of the two Hras alleles, as previously described [13]. *In vivo*, these cells will further be subjected to other deleterious mutations and a fraction of them will develop into invasive, metastatic skin tumors during the carcinogenesis process. The cells were a kind gift from Dr. Staurt H. Yuspa (National Cancer Institute, National Institutes of Health, Bethesda, Maryland, USA) [13].

Maintenance was carried in full Minimal Essential Medium growth media (Biological Industries, Israel) without Ca^2+^. Mouse Embryonic Fibroblasts (MEFs) were purchased from ATCC (SCRC-1008). Culturing was done in full Dulbecco Minimal Essential Medium (Biological Industries, Israel). Human ovary adenocarcinoma NAR (NCI/Adr-Res) cells were provided by M. Liskowitz (Weizmann Institute of Science, Israel) and are also part of the NCI 60 cell lines http://cancer.sanger.ac.uk/cell_lines/cbrowse/nci [14]. Culturing was carried in full RPMI 1640 (Biological Industries, Israel) growth media. All growth media contained 10% fetal calf serum, Penicillin (100 U/ml), Streptomycin (0.1 mg/ml), Nystatin (12.5 U/ml), and L-glutamine (2 mM) all from Biological Industries, Israel. Cells were cultured in humidified atmosphere at 37°C and 5% CO_2_.

### FRET Measurements

0.8 µM of the anchor, probe and targets oligos were dissolved in PBS in a black 96 well plate (In vitro scientific, Sunnyvale,CA, USA) and fluorescence intensity was measured at 37°C using a Synergy HT plate reader (Biotek, Winooski, USA). Three filters were used for detection of donor, acceptor and FRET signals [Excitation/±nm,Emission/±nm] : [485/20,528/20], [590/20,645/40], [485/20,645/40].

### *In vitro* Binding Assays

Probe specificity was assessed by detecting the FRET signal following hybridization to two 40 bp synthetic RNA oligonucleotide targets corresponding to the mutant and wt alleles as described in Table 1.

**Table 1:** Hras oligo probes sequences and modifications.

Oligonucleotide	Sequence (5′ –3′)
**1. HRAS-anchor-AF488 PS**	C*C*C*G*C*A*U*G*G*C*A*C*U*A*U*A-AF488
**2. HRAS-T-TR-8mer -PS**	TR-U*C*T*A*G*A*C*C
**3. HRAS-A-TR-8mer -PS**	TR -U*C*T*T*G*A*C*C
**4. HRAS-T-AF594 -8mer-PS**	AF594-U*C*T*A*G*A*C*C
**5. HRAS-A-AF594 -8mer-PS**	AF594-U*C*T*T*G*A*C*C
**6. HRAS-anchor-AF488 PSEnd**	SpC3-C*CCGCAUGGCACUAU*A-AF488
**7. HRAS-T-TR -8mer-PSEnd**	TR-U*CT**A**GAC*C
**8. HRAS-A-TR -8mer-PSEnd**	TR-UCT**T**GAC*C
**9. HRAS-U-targ RNA**	acagcagguc**u**agaagaguauagugccaugcgggaccagu
**10. HRAS-A-targ RNA**	acagcagguc**a**agaagaguauagugccaugcgggaccagu

Key: **A,C,U/T,G –**2′OMe RNA**, a,c,u,g –** RNA**, A,C,T,G** - LNA residues**, Bold font -** SNP site**, TR –** Texas Red**, AF –** Alexa Fluor, “*” - phosphorothioate **(PS)** internucleotide linkage**, PSEnd-** incidates phosphorothioate bonds only on terminal linakges of the molecule**, targ-** target sequence.

200 nM of the anchor, probe and target oligonucleotides were annealed in a total volume of 10 µl in phosphate buffered saline (PBS, 10 mM Na_2_PO_4_H_2_O (Merck), 2 mM K_2_PO_4_H_2_O (Merck) pH 7.4, 137 mM NaCl (Bio lab), 2.7 mM KCl (Merck) and 0.01% Triton (Santa Cruz) in DEPC treated water (Biological industries, Israel). Samples were analysed by fluorescence measurements taken every 0.1°C over a gradual temperature increase from 37°C to 75°C on a Roche Lightcycler® 480 (Roche Applied Sciences, Indianapolis, IN, US). The ROX (577/603) filter was used to detect signal from the Texas Red (583/607) Acceptor Probe and the FAM (492/518) filter was used to detect signal from the Alexa Fluor 488 (493/517) Donor Anchor. Base fluorescence levels of both oligo probes were calibrated to baseline according to 200 nM of each oligo so that increases in fluorescence above this level would account as energy transfer.

Probe binding to endogenous RNAs were performed in similar fashion. RNA was isolated from 308 and MEF cells using EZ-RNA isolation kit (Biological Industries, Israel) according to manufacturer instructions. 1 µg of the extracted RNA was incubated in a 96 well plate along with 200 nM of anchor and probe T in PBS (−/−) as described.

### Folding Prediction

Hras mRNA folding predictions were obtained using the mfold server (The RNA Institute, University at Albany, State University of New York).

### Hras Mutation Analysis

Genomic DNA was isolated from cell lines using a DNA extraction solution (Epicentre, Madison, WI, USA). 1 µg of DNA was subjected to polymerase chain reaction (PCR) analysis for Hras amplification using PrimeSTAR™ polymerase (Takara, Otsu, Japan) according to manufacturer instruction. Primers sequence was as listed below:

*Forward*: ctatagaggtgagctctgcctacc

*Reverse* : ctacattgaaacatcagccaagac

To confirm Hras mutation, restriction digestion was carried using XbaI restriction enzyme (Takara). 1 µl of XbaI enzyme was mixed with 2 µl 10x buffer, 0.1% BSA, 5 µl ultra pure water (BI) and 10 µl of purified DNA. After 1 h in 37°C the total volume of the reaction mix was loaded on 1% agarose gel (Hispanagar), stained with ethidium bromide (Bio-lab) and analyzed by gel electrophoresis. Bands were visualized using Gel doc™ XR molecular imager (Bio-Rad, Hercules, CA, USA). Hras replicon size was 700 bp and as a result of Hras mutation in codon 61 the total amplicon is expected to yield 2 restriction fragments 300 and 400 bp in size.

### Sequencing

PCR products were purified using PCR fragments extraction kit (Real-Biotech, Banqiao city, Taipei, Taiwan) and DNA concentration was measured using Nanodrop spectrophotometer (Thermo Scientific, Wien, Austria). 70 ng DNA was added to 10 pmole forward primer and 16 µl DDW. Sequencing was carried out using the ABI 3500xl Genetic analyser (Life Technologies, Carlsbad, CA, USA).

### Microroporation

Microporation (20) was conducted using Neon™ transfection system (Life Technologies, Carlsbad, CA, USA). Each sample contained 150 k cells, spun down for 5 min in 0.4 G. Then cells were placed on ice. Supernatant was removed and a reaction mixture containing 10 ul buffer T, 3 µM of each oligo as well as 7 µl PBS were added in the dark. Cells were microporated and immediately placed on ice. The following parameters were used for the microporation: pulse power - 1400 Volt/pulse width - 20 ms/pulse number - 1. After the microporation four identical samples were united into fresh tube containing full growing medium (without antibiotics), spun down and resuspended in 3 ml PBS containing 0.5 ml FCS.

Cells were plated on cell culture dishes with glass bottom (Grenier, Frickenhousen, Germany) that were pre-coated with collagen (BD, New Jersey, USA) for increased adherence. The coat was obtained by adding 10 µg/ml collagen solution to each plate, 1 h incubation in 37°C and two 5 min washes of the plate with PBS. After plating, cells were kept in 4°C until confocal analysis was done.

### Acceptor Photobleaching Assay using Confocal Live Cell Imaging

The FRET donor AF488 label and the acceptor TR label were detected in living cells in a single cell resolution by Zeiss LSM 510-META confocal microscope (Carl Zeiss MicroImaging GmbH, Jena). The cells were kept in a growing chamber in 37°C, 5% CO_2_ throughout the assay. Acceptor photobleaching was performed only in cells that visibly contained both fluorophores. AF488 and TR excitation were performed using 488_nm_ and 561_nm_ lasers, respectively and fluorescence detection was carried out using BP 505–550 and LP 575 detection filters. Photobleaching intensity (Red burn) was 400IT. Red burn intensity was measured accordantly with green fold change in fluorescence.

### FRET Calculation and Statistics

#### Plate reader measurements

FRET can be detected by exciting the labelled specimen with light of wavelengths corresponding to the excitation spectrum of the donor and detecting light emitted at the wavelengths corresponding to the emission spectrum of the acceptor. There is a need to correct several potential distortions during FRET measurements. These alterations include any detection of donor fluorescence with the acceptor emission filter and any detection of acceptor fluorescence with the donor emission filter as well as the dependence of FRET on the concentration of the donor and acceptor. In this assay, we used three filter sets to measure FRET, thus here we used the following formula in order to correct the above distortions. It should be noted that different equations exists for FRET calculation [15] and we did not find significant difference in the results trend between them.
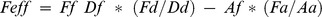



Capital letters represent the filter used (F-FRET, D- donor, A-acceptor) and small letter represents the fluorophors present in the sample (F- both donor and acceptor, D- donor, A-acceptor).

- *F*eff - FRET effective,
- *Ff* - The signal received using the FRET filter set from specimen where both fluorophors present.
- *Df* - The signal received using the donor filter set from specimen where both fluorophors present.
- *Fd*- The signal received using the FRET filter set from specimen where only donor fluorophor is present.
- *Dd-* The signal received using the donor filter set from specimen where only donor fluorophor is present.
- *Af-* The signal received using the Acceptor filter set from specimen where both fluorophors are present.
- *Fa-* The signal received using the FRET filter set from specimen where only acceptor fluorophor is present.
- *Aa* - The signal received using the Acceptor filter set from specimen where only acceptor fluorophor is present.

#### Confocal microscopy

Acceptor photobleaching assay measures the increase in green donor emission while red acceptor emission is decreased by photobleaching (sensitized emission). During the course of the photobleaching both the green and the red signals are measured every few seconds before and after the red photobleaching. In this case, since the extent of red decay influences the increase in the green donor intensity, FRET level is corrected accordantly, and is calculated as the ratio between the level of donor green increase and the acceptor red decay as follows:
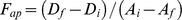



**Whereas,** *F_ap_ –* FRET Acceptor photobleaching, *D –* donor signal, *A –* acceptor signal, *f-* final signal (after acceptor photobleach), *i–* initial signal (before photobleach).

Green signal before and after photobleaching was calculated as the mean of two measurements respectively. Samples were analyzed using LSM image browser software (Carl Zeiss MicroImaging GmbH, Jena) for green and red intensities and FRET output was calculated as indicated. One sample *t*-test was carried on each result before being included in the sample average. One tailed unpaired *t*-test was used to test the significance between samples mean with p-value of 0.05. Statistical analysis was carried using the Statistical Product and Service Solutions (SPSS) data editor software (IBM, NY, USA).

## Results

### Design, Synthesis and Optimization of FRET Probes

A variety of probe sequences were tested to achieve optimal SNP discrimination. Several spacing variants between the anchor and the specific probe binding sites were also tested to achieve optimal FRET. Previous work have shown that the optimal distance varied with FRET pairs and that FRET pairs with closer emission/excitation wavelengths did better farther apart and those with spectra far apart did better when positional spatially closer. For FAM-ROX (the closest FRET pair to AF488-TexRed used in this study), 5–8 bases spacing was found optimal [16]–[18]. In our system a spacing of 4 bases proved to be the most effective inter-probe spacing to maximize FRET signal. Thus for the “spectrally further apart” AF488-TexRed our findings are reasonable and consistent with the prior art (Figure S1).

We synthesized and tested several variants of the chemically modified FRET oligos to optimize specificity. The chemical modifications employed are shown in Figure 2. The different probe sequences and modification patterns are detailed in Table 1.

The Anchor (Oligo 1) and Allele-specific Probes (Oligos 2–5) were made using PS bonds at all internucleotide linkages to protect the oligonucletoides from nuclease degradation. However, the PS modification makes the oligonucleotides more hydrophobic and increases non-specific interaction with proteins, which can cause toxicity and may influence intracellular distribution (20). Further, the PS modification reduces binding affinity (lowers T_m_). We therefore also tested variants having PS internucleotide linkages only between the terminal residues (Oligo 6 - Anchor; Oligos 7 and 8 - Allele-specific Probes,). 2′OMe residues are susceptible to exonuclease attack but are fairly resistant to mammalian endonucleases [19]. The variants with minimal PS modification (Oligos 6–8) had PS bonds at their terminal linkages as well as a dye or C3 spacer at the ends, which will confer protection from exonuclease attack and therefore should be stable when transfected into cells.

The Anchor (Oligos 1 and 6) had a 3′-AF488 dye which served as the FRET donor. The Allele-specific Probes had either a 5′-TR dye (Oligos 2 and 3) or a 5′-AF594 dye (Oligos 4 and 5) which served as the FRET acceptor. When hybridized to the Hras target mRNA, the 3′-end of the Anchor oligo (FRET donor) will be positioned near the 5′-end of the Allele-specific Probe (FRET acceptor) in an ideal configuration for FRET to occur. The same oligonucleotides free in solution or bound individually to different targets will not be close enough to one another for FRET to occur. When annealed to the same target, excitation of the donor at around 488 nm will lead to fluorescence emission at around 520 nm, transferring energy to the acceptor dye, which will in turn emit a fluorescence signal around 615 nm (Figure S2). The red dyes are poorly excited by light at 488 nm [20], so red fluorescence emission should only be detected when FRET occurs, which is dependent upon both the donor and acceptor dyes being in close spatial proximity.

For the purpose of testing the specificity of oligos *in vitro*, two 40 bp RNA oligonucleotides were synthesized and served as synthetic targets of the probes. Target U oligo was the perfect-matched target of probe T, and represents a short fragment of mutant Hras allele, while target A is a single mismatched target to the T probe which is a part of the wt Hras allele. Similarly, probe A is a perfect match to target A oligo and a single mismatch to the target U oligo.

**Figure 3:**
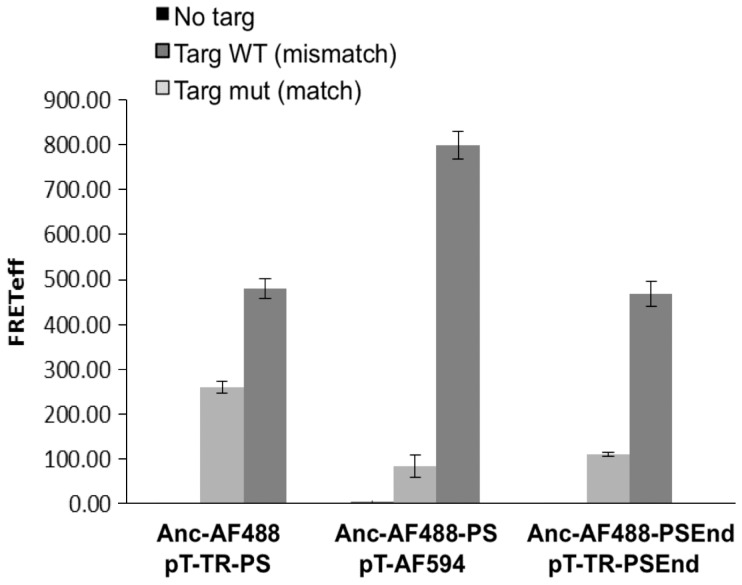
Influence of chemical modifications on target specificity and FRET intensity. Three differentially modified FRET couples were incubated with: no target (left bar), mismatch target (middle bar) or full match target (right bar) and FRET was measured. Targ – target, Anc - Anchor Alexa Fluor 488 (AF488), PS- ODNs heavily modified with PS bonds, PSEnd- PS modifiIcation only at the edge of the ODN, pT- mutant specific probe T marked with either Texas Red (TR) or Alexa Fluor 594 (**AF594**) as acceptor fluorophore.

When comparing mismatch discrimination between the different versions of FRET pairs (Figure 3), Texas Red labled oligos showed lower FRET signal in match target than AF594 labled probes. Ultimately, it can be concluded that the probes that were heavily modified with PS bonds and contained AF488 as FRET donor and AF594 as FRET acceptor showed the highest FRET signal and the strongest discrimination efficiency between match and mismatch target. This implies that it is possible to discriminate between perfect match and mismatched target in 37°C while changes in the oligos modification design and labelling can highly influence the signal level observed.

### Probes Compete for Binding with mRNA Secondary Structure

It is well known that a functional mRNA molecule in a living cell always has RNA-binding proteins on it, forming a ribonucleoprotein (RNP). Furthermore, an mRNA molecule often has double stranded portions and forms secondary (folded) structures. Although predictions of mRNA secondary structure can be made using software such as *mfold* (http://www.bioinfo.rpi.edu/applications/mfold/old/dna/), they may be inaccurate due to limitations of the biophysical models used, and the limited understanding of protein-RNA interaction [4]. The length of murine Hras mRNA is around 2 Kbp (depending on the splice variant) thus, it will preferably fold into an energetically favoured secondary structure.

Hras folding predictions include several optional secondary structures, ranked in an ascending order of free energy ranging from ΔG = −315.25 up to −306.20 Kcal/mole.

**Figure 4:**
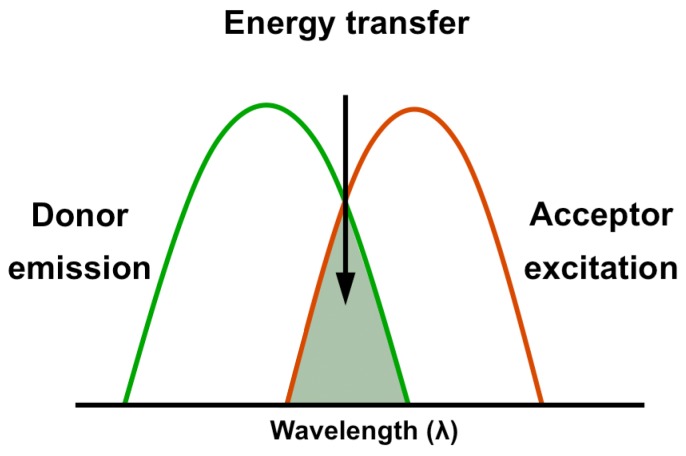
Secondary structure prediction of Hras target sequences. Predicted folding state of Hras *synthetic targets* are shown in structures (**A–C**)**.** Green underline - anchor binding site. Red underline - Probe binding site. Yellow frame - codon 61. (**A**) and (**B**) represent the wt A allele whereas C represents the U mutant allele. The 40 bp synthetic target sequence is predicted to create a hairpin structure under physiological conditions. Structure (**D**) represents the energetically favored structure of *endogenous* Hras mRNA. According to this structure, the mutated codon is in a hairpin structure.

In 8 out of 12 structures, including the energetically favoured structure (4D), the probe binding site, including codon 61 (mutation site), is located on the edge of a loop, in positions 384–410. It is unknown whether the probe can bind to the free bases in the loop structure while it is in its folded state or whether the transcript secondary structure opens in the presence of the complimentary high affinity FRET pair. The oligos with modified bases provide higher affinity towards the target sequence than intramolecular complementary unmodified sequences, therefore, probably competition occurred. We next investigated whether the mRNA was accessible for binding in spite of its secondary structure (Figure 4).

### SNP Discrimination *in vitro*

**Figure 5:**
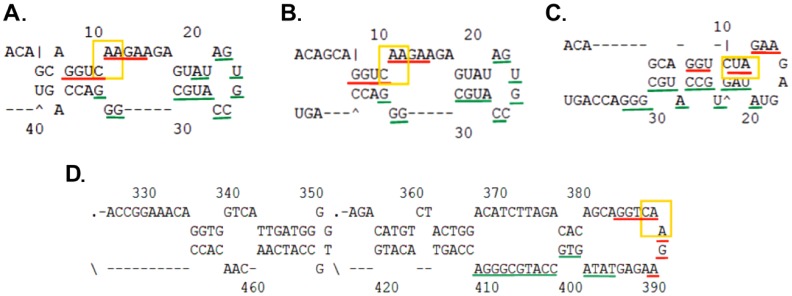
A FRET pair contains two fluorophores with overlapping spectrum. When the donor is excited, FRET enables the fluorescence emission of the acceptor. It should be noted that the background noise arising from the leakage between the donor and acceptor channels need to be deducted.

**Figure 6:**
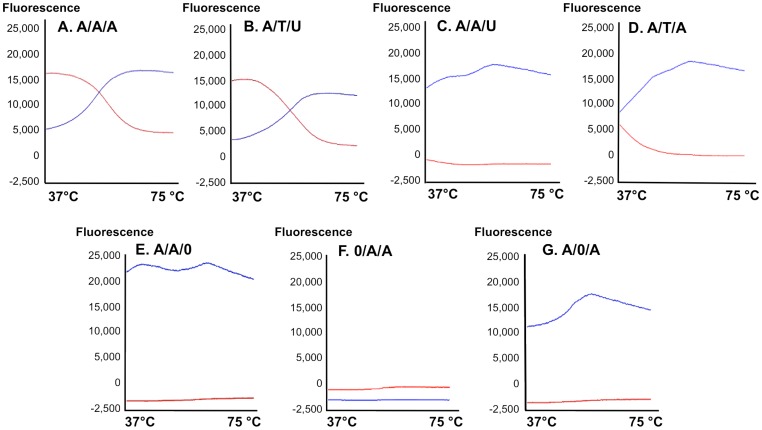
FRET probes are SNP specific *in vitro* at 37°C. Multicomponent plots indicating fluorescent signals from the anchor (blue line) and the allele specific probe (red line) as a course of temperature increase (37–75°C). **A** – Anchor/**A, T** allele specific probe or O – no probe/**A, U** – target RNA oligo complement to A and T probes respectively. Perfect match between the probe and target results in an energy transfer between the donor anchor and acceptor probe. When both the donor anchor and acceptor probe are bound to target at 37°C, their proximity causes energy transfer resulting in red fluorescence. As a course of temperature increase, the oligos denaturate from target (51°C), energy transfer ceases and only the donor fluorescence can be detected, causing a typical “8” shaped FRET plot to be generated (**A, B**). A single mismatch in target sequence results in low affinity of the oligos therefore no FRET response is detected (**C, D**). No target control (**E**), no donor control (**F**), no acceptor control (**G**).

Next, we wanted to validate that the signal observed through the emission of the acceptor fluorophore represents a true FRET signal rather than leakage from the emission spectra of the donor or excitation of the acceptor. Moreover, calculation of FRET through measuring the light emitted from the acceptor after excitation of the donor (Figure 5) can be made using different formulas [15]. Therefore, measuring energy transfer in real time would be a validated assay. To this end, we utilized a qPCR machine as a fluorescence measurement device that enabled plotting each of the fluorophores emission intensity throughout the assay. Figure 6 shows plots of the relative change in fluorescence compared to the baseline fluorescence at the beginning of the experiment. Introduction of a fully matched target to both ODN results in cooperate binding to target and strong emission of the acceptor as a result of donor proximity leading to energy transfer in 37°C. As temperature increases the probe and anchor unwind from target, causing energy transfer to cease; allowing the red fluorescence to decrease and the green signal to increase (A,B). The multicomponent plot indicates the probe denatures from its target sequence at 51°C (indicating its T_m_). When both oligos are placed with a single mismatch target sequence, the affinity towards the target is not sufficient to obtain both ODN bound to the target, abolishing FRET (C,D).

When both anchor and probe are present with no target, the donor green signal is detected while no signal is generated by the red acceptor, thus no FRET has occurred (Figure 6E). In the absence of green anchor no signal is detected due to absence of FRET donor (F) whereas absence of red acceptor probe gives rise only to green fluorescence (G).

### Detection of Hras Point Mutation in Endogenous RNA

**Figure 7:**
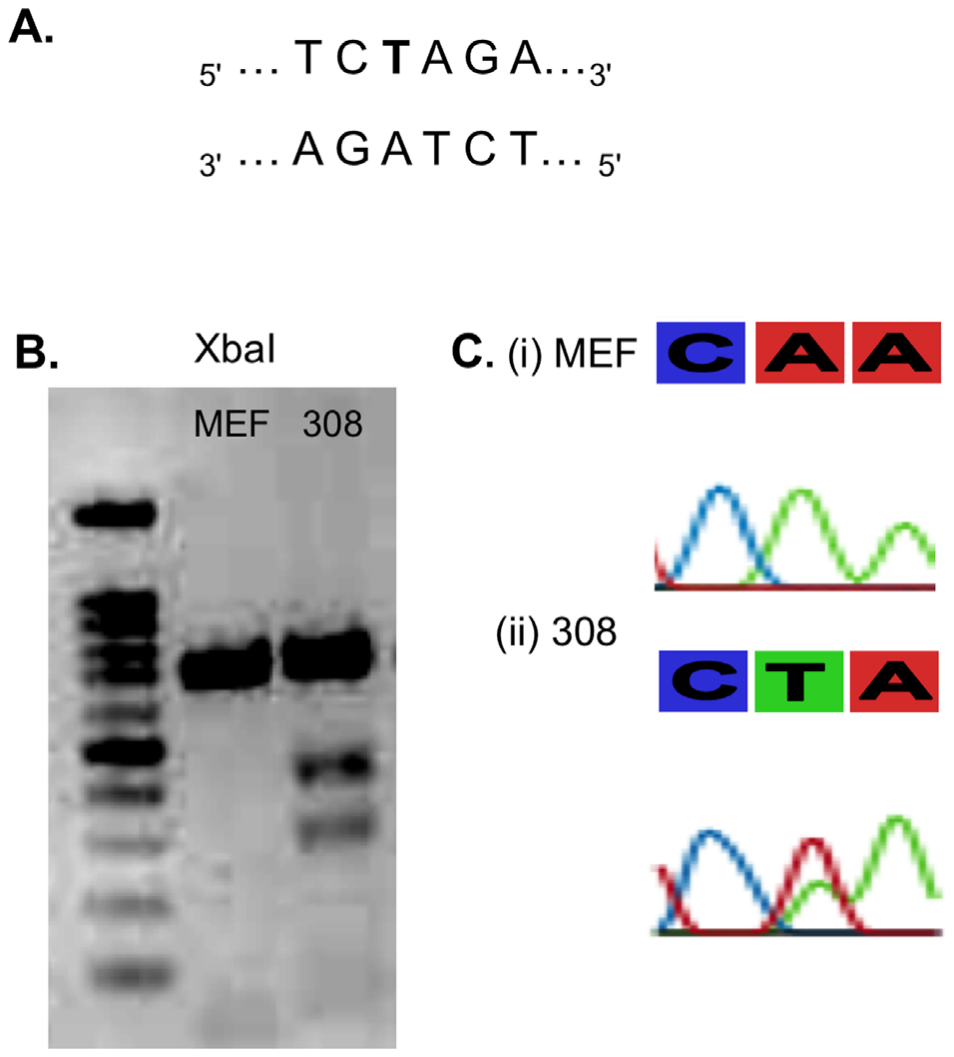
Point mutation in Hras gene in DMBA/TPA treated cells. (**A**) The A>T substitution causes the appearance of XbaI consensus site (**B**) XbaI digestion of Hras in MEF (left lane) and 308 cells (right lane). Two restriction fragments of 300 and 400 bp emerge in mutated Hras resulting from cleavage of full length 700 bp replicon in the newly formed XbaI restriction site. (**C**) Sequencing of Hras codon 61 reveals A>T transversion in of 308 cell line which is derived from early DMBA/TPA induced papillomas **(ii).** Mouse embryonic fibroblasts (MEFs) hold the typical A allele **(i)**.

**Figure 8:**
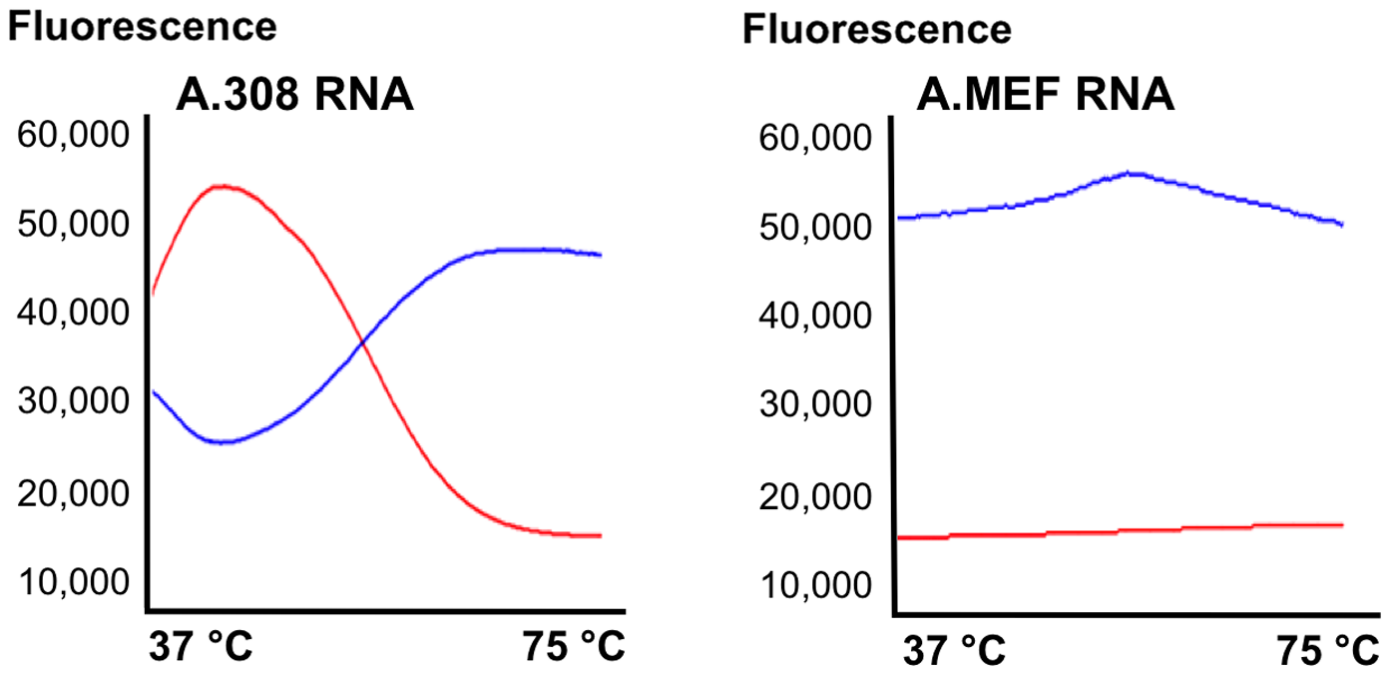
Point mutation detection in endogenous Hras by FRET probes at 37°C. Total RNA was extracted from 308 mutated cells and MEF cells prior to FRET measurement. **A**. In 308 cells, Mutation specific probe (T) (TR, PSEnd modified) was fully complementary to the mutant allele thus FRET occurred and red emission was observed at 37°C. As cycles progress, the temperature increases and the probes unwind from their target thus energy transfer ceases causing the typical “8” plot to be formed. **B**. In MEF cells, donor excitation did not cause energy transfer to the FRET acceptor because the mutant T probe did not bind the wt Hras mRNA.

Ras is a frequently mutated oncogene in human cancer [21]. Mutations that permanently activate Ras are found in 20–25% of all human tumors and up to 90% in certain types of cancer [21]. In our current model of skin SCC, point mutation in codon 61 of Hras is one of the first genetic events triggering tumor formation [22] **.** The Hras point mutation in codon 61 of 308 cells causes appearance of XbaI restriction site (Figure 7A) thus the 700 bp PCR fragement was cleaved to 300 bp and 400 bp restriction fragements (B). The mutation was confirmed by sequencing (C).The mutated DNA is transcribed to mutant mRNA and these transcripts are found in multiple copies in each cell. Mostly, steady-state levels of mutated transcripts of key-genes as Hras are elevated during tumorgenesis [23], thus increasing target concentration and detection chances. RNA from 308 and MEF cells was extracted and subjected to FRET analysis using the qPCR platform as done with synthetic RNA targets. Figure 8 shows that the mutant allele specific FRET pair was able to specifically identify the point mutation generating the typical “8” plot symbolizing a FRET response. At the beginning of the assay, at 37°C, both the donor and acceptor ODNs are bound to target, thus the red emission is detected. Once reaching its T_m_, the probe detaches from target and only the anchors’ green fluorescence is observed (A). In MEF cells however, FRET response was not detected, due to the single mismatch between T probe and the wt target mRNA.

### Detection of SNPs in mRNA in Living Cells

Once entering the dynamic cellular environment, there are numerous challenges facing the FRET oligos before they can bind their target in a specific and functional manner. The ODNs need to be stable and non-degradable. They are also expected to independently locate and bind their specific target mRNA, which might be folded, wrapped with binding proteins, located in p-bodies or is being translated at a given time point. The FRET pair oligos were transfected into three cell lines representing various allelic combinations.

NAR – human, lacking the murine Hras mutation - these cells served as positive control to the target since they were transfected with the synthetic 40 bp target. It should be noted that due to the fast kinetics of the probes (5∼min) it was possible to visualize the binding (FRET) event before the un-modified target ODN was degraded.

MEF – murine cells, containing only the wt (A) alleles (match to A/A and mismatch to A/T FRET pair).

Finally, the 308 murine cells, containing one mutant Hras allele and one wt allele (one allele is a full match to the A/T and one to the A/A FRET pair).

**Figure 9:**
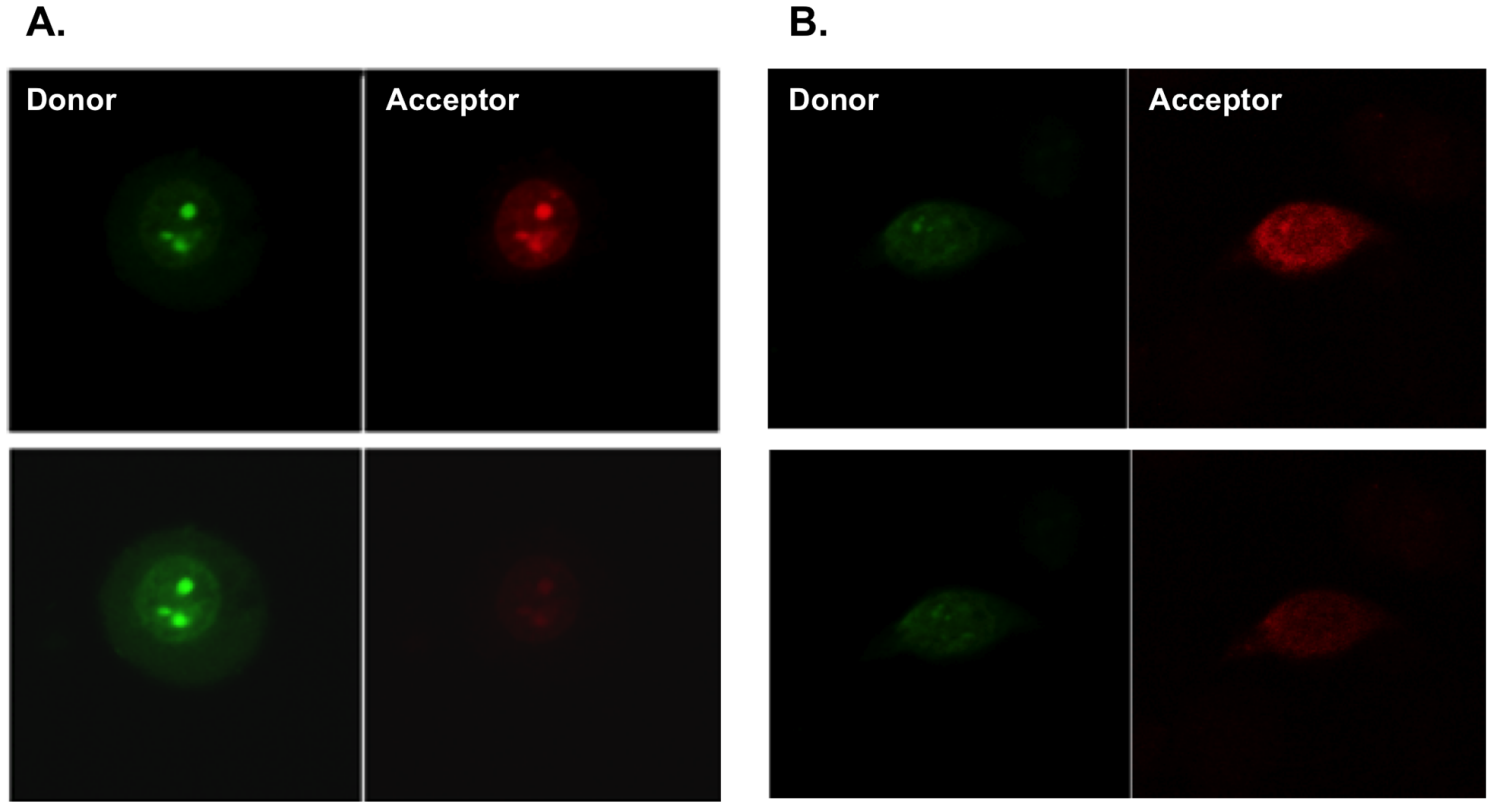
Single cell Acceptor photobleach analysis. The Anchor (FRET donor, AF488) and mutant Probe (FRET acceptor, TR) were co-transfected to the same cell and fluorescence was measured before (upper panel) and after (lower panel) red photobleaching. As a result of photobleaching, the acceptors red signal decreases. When energy transfer occurred, the donor green emission increased after the acceptors photobleach. **A.** In 308 cells, which endogenously carry the Hras point mutation, an increase in green donors signal was observed after acceptor photobleach whereas in **B.** Hras wt MEF cells, no increase in donors signal was observed.

Single cells were subjected to acceptor photobleaching (AP) while placed in an incubation chamber during confocal microscopy. Representative images are shown in Figure 9– AP assays measure an increase in the green donor signal after the red acceptor is photobleached. The increase in donor signal is reflective of the energy transferred to the acceptor before it was photobleached, therefore indicative of a FRET response (Figure 9A). Zeiss LSM image browser software was used for fluorescence quantization before and after acceptor photobleach.

**Figure 10:**
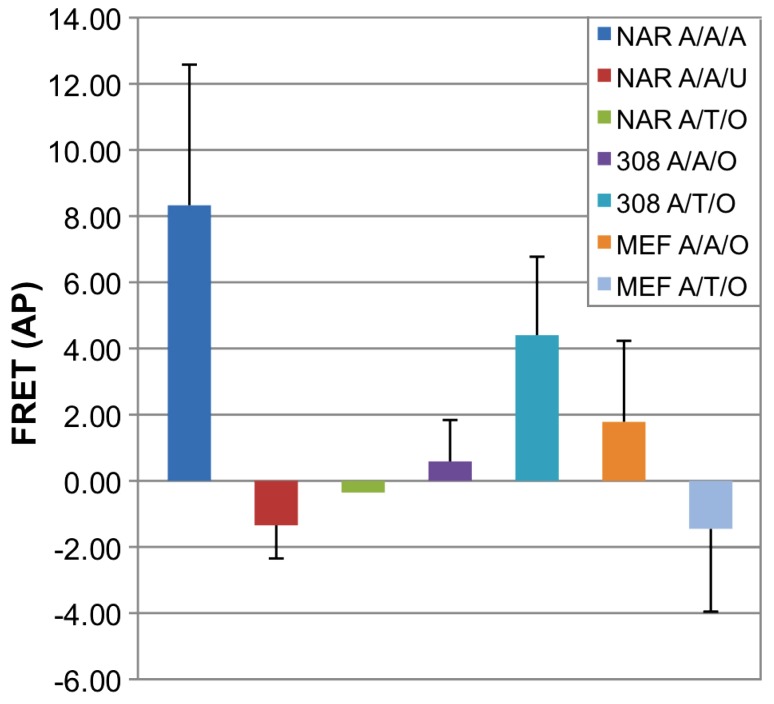
Point mutation detection by Hras allele specific FRET probes in NAR, 308 and MEF cells. A - anchor**/**A,T **-** allele specific probe**/**A,U synthetic target oligo or no (O) target. After oligos transfection, cells were suspected to Acceptor Photobleach (AP) analysis. NAR cells containing Anchor and wt (A) probe showed relatively high FRET with full matched A synthetic target (blue left bar). A single mismatch in target sequence showed no FRET (U, red bar). When no target was present in the cells (NAR, green bar), no FRET was observed, indicating no FRET background. WT specific (A) probe induced a positive FRET response in both 308 and MEF cells, indicating presence of the wt allele (308– purple bar, MEF - orange bar). Mutant T probe exhibited positive FRET response only in 308 mutated cells (turquoise) as opposed to wt MEF cells (light blue).

Average values of FRET signals were calculated as the ratio between the donor signal increase and the acceptor decrease and are summarized in Figure 10. The FRET pair lacking exogenous/endogenous target (NAR A/T/O) did not give rise to a FRET signal, implying there is no unspecific binding of the ODNs to other targets in the cell. In human NAR cells containing synthetic target ODN, a single mismatch in target sequence showed no FRET whereas fully matched probes displayed high FRET signal.

Accordantly, a positive FRET response was observed both in MEF and 308 cells when transfected with A probe (Figure 10 - purple and orange bars). Transfection of T probe, on the other hand, gave rise to increase in donor signal after photobleaching (positive FRET) in 308 cells but not in MEF cells, reflecting the fact that Allele T only exists in 308 cells and not in MEF cells.

The AP assay was preformed immediately after transfection due to fast hybridization of the probes to their target. Throughout a time course of 10 min - 24 h no significant change in FRET intensity was observed.

## Discussion

Local genetic aberrations often lead to malignancies as tumorgenesis and other malfunctions. The damage could range from single point mutations up to large chromosomal rearrangement [24]. An increasing number of jeopardizing genetic alterations is already known and is used today for *ex vivo* genetic screens on fixed cells and as biomarkers for prognosis and disease management. Methods for real time monitoring of genetic alterations in live cells offer tremendous opportunities for biological and disease studies. It could serve as a powerful tool for detection and diagnostics and will significantly impact drug discovery and medical diagnostics including shortening the duration of genetic tests procedures.

Currently available technologies for gene detection in intact cells such as in-situ hybridization (ISH) cannot be used *in vivo*, since ISH studies rely on washing out unbound ODNs to reduce background signal. To perform gene detection in living cells, the probes must be able to recognize the target with high specificity, convert target recognition directly into a measurable signal with high signal-to-background ratio, and allow for differentiation between real and false-positive events. Probes must also be delivered into living cells with high efficiency and specificity. So far, several methods for real time mRNA detection based on labelled antisense molecular probes were developed, exploiting the progress in manipulations of nucleic acid that allow *in vivo* utilization of the probes thank to increased nuclease resistance and target specificity. These previous strategies [2], [5] have shown the use of modified ODN for the purpose of detection of a specific endogenous mRNA in the cell, proving feasibility of the approach on natural, folded, protein engaged mRNA in living cells.

In the present study, we have challenged the molecular probes by constructing allele-specific probes aimed not only to identify a certain type of transcript but to discriminate between two alleles differing in only one nucleotide. Therefore, calibrated and selective design of ODN was a crucial step, determining the final outcome of the following studies.

Both FRET pair ODNs were modified with 2′OMe residues, phosphorothioate linkages and LNA residues at the SNP site to improve allele discrimination. Use of DNA probes would form a DNA:RNA heteroduplex between the ODNs and the Hras mRNA target, which could lead to RNase H mediated degradation of the mRNA, an undesired outcome [7]. 2′OMe RNA shows an increase in binding affinity to an RNA target compared with DNA probes. The LNA modification shows an even greater increase in binding affinity. Neither 2′OMe RNA or LNA bound to RNA leads to RNase H degradation. Further, incorporation of these chemical modifications enables use of very short probes (in this case, 8mers) which improves mismatch discrimination. In particular, positioning three LNA residues immediately at the SNP site maximizes the ΔT_m_ between match and mismatch [12]. Since thermodynamic parameters do not exist to predict T_m_ using these modified bases against an RNA target, we empirically tested the influence of the different designs (referring to length, chemical modifications, and inter oligos spacing) on probes functionality. Each pair underwent multiple rounds of selection hybridization to achieve optimal discrimination under physiological conditions. Then the different designs of the FRET pairs were compared as shown in Figure 3. As expected, none of the pairs showed any FRET background signal in the absence of the target nucleic acid. FRET pairs made using fluorescein as the donor (in place of AF488) showed low signal, probably due to the lower quantum efficiency of fluorescein at elevated temperature of 37°C compared with AF488 (data not shown). In addition, the anchor ODN with fluorescein exhibited higher background signal in the FRET optical setting when the pairing probe was present and target RNA oligo was absent. It’s reasoned that fluorescein labelled probe may have higher non-specific interaction with the TR or AF594 labelled probe than that of AF488, thus generating higher FRET background signal. Within the set of sequence variants tested here, all three FRET pair exhibited discrimination in FRET signal in favour of the match target whereas the full PS modification together with AF594 as acceptor (and AF488 as donor) worked best to maximize allelic discrimination at a fixed temperature of 37°C. In many molecular biology assays, adjusting the temperature of the hybridization reaction is done to maximize the ΔT_m_ between match and mismatch. In this case, temperature is fixed at 37°C, so adjustments can only be made on the oligo sequence/length and choice of chemical modifications employed.

For specific detection of the desired transcript, the targeted segment has to be unique, such that it does not have significant sequence homology with other RNA sequences and also it should not have a structural constraint that may prevent hybridization of the probe. It is well known that RNA molecules within living cells are commonly associated with RNA-binding proteins, e.g., ribonucleoproteins (RNPs) and form complex secondary structures. It is preferable to avoid targeting RNA sequences that are double stranded, or occupied by RNA binding proteins; Since the models offered by the different prediction software are often inaccurate, the ability to reliably predict the mRNA folding state and binding proteins is limited [5]. As suggested by our folding query, the endogenous Hras folds into a secondary structure and creates a stem and loop in the probes binding site, forcing the probes to compete with the RNA strand or the RNA-binding protein in order to hybridize to the target. Our results show binding of the probes to short synthetic RNA target as well as to long endogenous Hras RNA outside and inside living cells. This led us to conclude that the ODN probes compete with target mRNA intra-molecular base-pairing thanks to their backbone modifications. The 2′OMe and LNA modification provide the ODNs with higher affinity (higher T_m_) and improved specificity toward the target [25]. In case the intramolecular complementry sequence is loosley bound, i.e bound through a small number of basepairing, as implied by its folding prediction, the target RNA will gain more energy during duplex formation to the ODN than to its complementry, unmodified sequence. This will lead to the mRNA secondary structure to untie and the ODNs to bind. Moreover, these probes are not degraded, stay functional and bind their target sequence *in vitro* and in living cells under physiological conditions, and most interestingly, can discriminate between two alleles differing in only one nucleotide.

As was observed before, ODN probes rapidly enter the cell nucleus after entering the cytoplasm, and specific hybridization patterns can be observed within 1–2 minutes irrespective of the target RNA [26]. Moreover, it was shown that linear antisense 2′OMe RNA ODNs could hybridize to nuclear RNAs, whereas unmodified linear antisense ODNs could not. In agreement with this previous work, our 2′OMe modified ODNs displayed fast kinetics and quick sequestration in the nucleus. When comparing the immediate reaction of linear modified ODN as in this case with the kinetic rate of the PNA-MB, which require 48 h incubation in transfected cells, there is a clear advantage to a quick and immediate reaction, which this platform provides to these diagnostic applications.

An optional way to prevent accumulation of antisense ODNs in the nucleus is by attaching macromolecules (e.g. NeutrAvidin) to the ODN so that they are too large to pass through the nuclear pores [27]. However, the presence of a macromolecule makes efficient cytosolic delivery of these probes challenging due to the big size of the transfected complex [28]. In spite of the quick nuclear sequestration we managed to see FRET signal in the cells without macromolecule conjugation and therefore did not modify the ODN in this way.

The qPCR provided a platform for *in vitro* FRET measurement to actually visualize the energy transfer between the FRET pair, rather than calculating it out of a fluorescent output which needs to be processed and “repaired” using various formulas different in their level of accuracy. *In vitro* have shown high specificity of our modified FRET pair towards both exogenous and endogenous targets at 37°C.

The differential FRET signal seen in living cells indicated that the ODNs survived the cells environment without being trapped in the endosomal pathway. The endosomal escape in our case is attributed to the method of transfection used, i.e. microporation, which was utilized due to previous testimonies in which it was said to contribute to this escape [28].

Harvey-ras gene is a highly potent oncogene, which was found to be one of the main prognostic factors along with c-fos, c-myc and p53 of breast carcinoma [29]. Mostly, a single mutated gene cannot independently predict cancer recurrence and patient survival but double or triple expression of proto oncogenes and tumor suppressors is required. However, the effect of individual oncogenes to predict survival was greatest for Ha-ras and c-fos [30]. As for other carcinomas, the value of each of the mutated proteins should further be investigated [31].

In conclusion, this work presents a non-invasive method that allows the detection of point mutation in living cells. This proof-of-concept study can be expanded for detection of genetic aberrations in clinical diagnostics as well as a research tool.

## Supporting Information

Figure S1 **Optimization between the Anchor (donor) and Probe (acceptor) oligonucleotides.** **(a)**: alignment of the anchor, probe, and target sequences is shown. **(b)**: FRET signal intensity comparison with 2, 3, 4, or 5 bases separation between the Anchor and Probe oligonucleotides. The Anchor oligonucleotide is the same as Oligo 1 in Table 1, except that the donor fluorophore is FAM (6-carboxyfluorescein); Probe = Oligo 2, Mismatch target = Oligo 10, and Match target = Oligo 9.
(PDF)

Figure S2 **Fluorescence resonance energy transfer demonstrated by fluorescence emission spectra.** 200 nM Anchor (FRET Donor, Oligo 1) and T-allele Probe (FRET Acceptor, Oligo 2) were incubated and measured at 37°C without target RNA (Blue), with mismatch target Oligo 10 (Green), and with match target Oligo 9 (Red).
(PDF)
