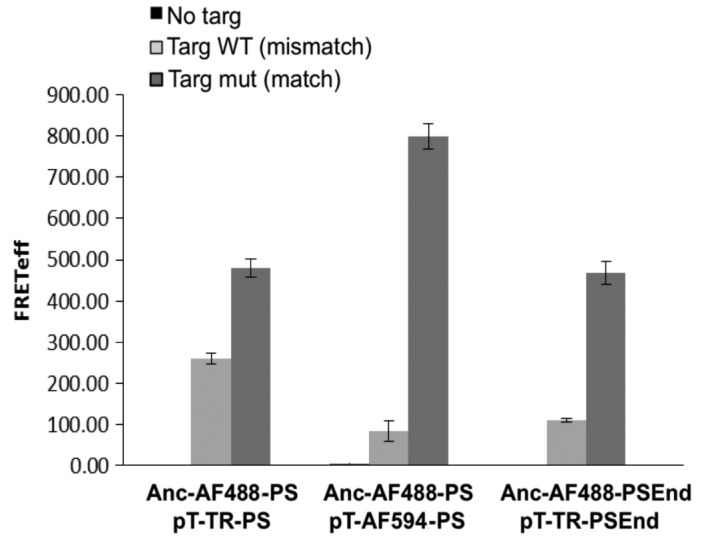# Correction: SNP Detection in mRNA in Living Cells Using Allele Specific FRET Probes

**DOI:** 10.1371/annotation/d41768c5-b58a-4355-ad17-166f70e347b8

**Published:** 2013-11-07

**Authors:** Liya Dahan, Lingyan Huang, Ranit Kedmi, Mark A. Behlke, Dan Peer

Figures 4 and 5 were incorrectly switched. The image currently appearing as Figure 4 belongs with the title and legend of Figure 5, and the image currently appearing as Figure 5 belongs with the title and legend of Figure 4. The titles and legends themselves are in correct order.

In addition, there was an error in Figure 3. Please see the correct Figure 3 here: 

**Figure:**