# Supplementary material for: SNP Detection in mRNA in Living Cells Using Allele Specific FRET Probes
Source: PLoS One. 2013 Sep 9;8(9):e72389. doi: 10.1371/journal.pone.0072389 (PMC3767744; doi:10.1371/journal.pone.0072389)
Supplement: Figure S1 — Optimization between the Anchor (donor) and Probe (acceptor) oligonucleotides. (a): alignment of the anchor, probe, and target sequences is shown. (b): FRET signal intensity comparison with 2, 3, 4, or 5 bases separation between the Anchor and Probe oligonucleotides. The Anchor oligonucleotide is the same as Oligo 1 in Table 1, except that the donor fluorophore is FAM (6-carboxyfluorescein); Probe = Oligo 2, Mismatch target = Oligo 10, and Match target = Oligo 9. (PDF) [file pone.0072389.s001.pdf]

```
Target      | 5'  acagcaggucAagaagaguauagugccaugcgggaccaguaca  3'
------------+---------------------------------------------------
            |           ::::::::    ::::::::::::::::
            | 3'        CCAGTTCTx   xTATCACGGTACGCCCT      5nt gap
            |
            |           ::::::::   ::::::::::::::::
Probe and   | 3'        CCAGTTCTx  xATATCACGGTACGCCC       4nt gap
Anchor      |
placement   |           ::::::::  ::::::::::::::::
            | 3'        CCAGTTCTx xCATATCACGGTACGCC        3nt gap
            |
            |           :::::::: ::::::::::::::::
            | 3'        CCAGTTCTxxTCATATCACGGTACGC         2nt gap
```

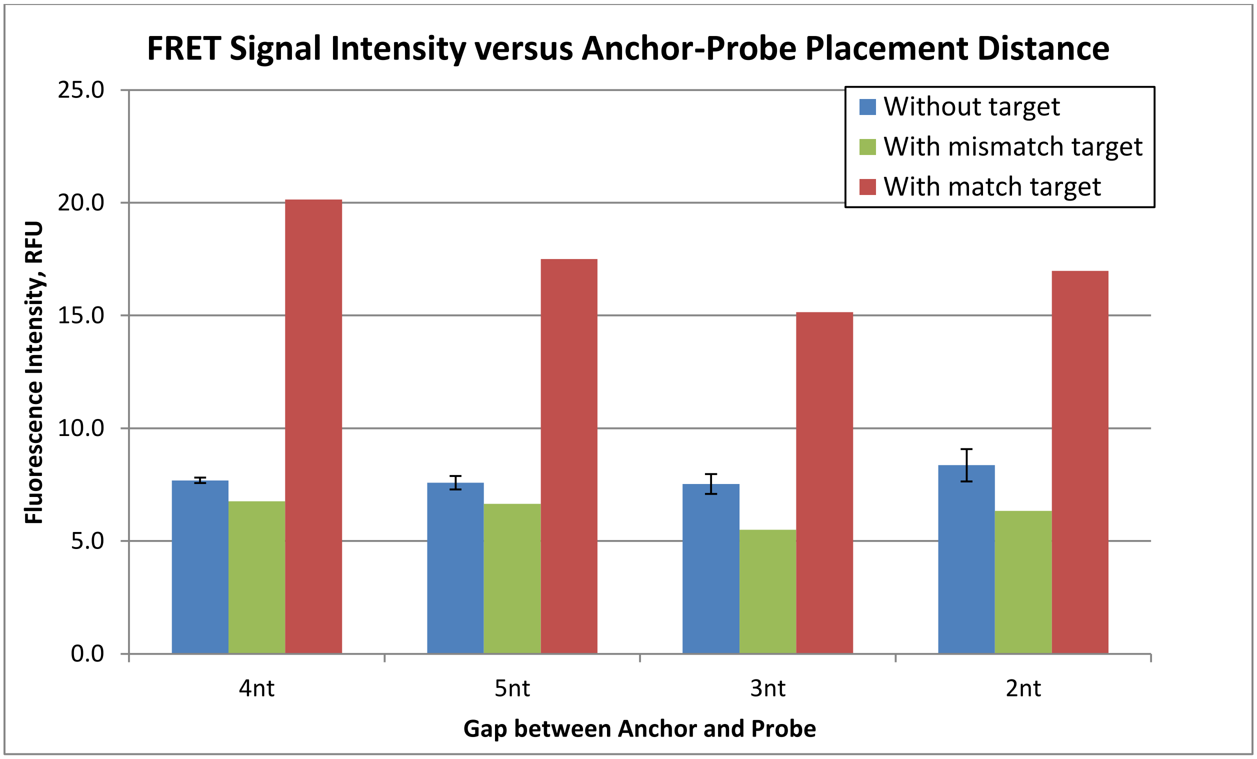

Figure S1. Optimization between the Anchor (donor) and Probe (acceptor) oligonucleotides. **(a)**: alignment of the anchor, probe, and target sequences is shown. **(b)**: FRET signal intensity comparison with 2, 3, 4, or 5 bases separation between the Anchor and Probe oligonucleotides. The Anchor oligonucleotide is the same as Oligo 1 in Table 1, except that the donor fluorophore is FAM (6-carboxyfluorescein); Probe = Oligo 2, Mismatch target = Oligo 10, and Match target = Oligo 9.
