# Supplementary material for: SNP Detection in mRNA in Living Cells Using Allele Specific FRET Probes
Source: PLoS One. 2013 Sep 9;8(9):e72389. doi: 10.1371/journal.pone.0072389 (PMC3767744; doi:10.1371/journal.pone.0072389)
Supplement: Figure S2 — Fluorescence resonance energy transfer demonstrated by fluorescence emission spectra. 200 nM Anchor (FRET Donor, Oligo 1) and T-allele Probe (FRET Acceptor, Oligo 2) were incubated and measured at 37°C without target RNA (Blue), with mismatch target Oligo 10 (Green), and with match target Oligo 9 (Red). (PDF) [file pone.0072389.s002.pdf]

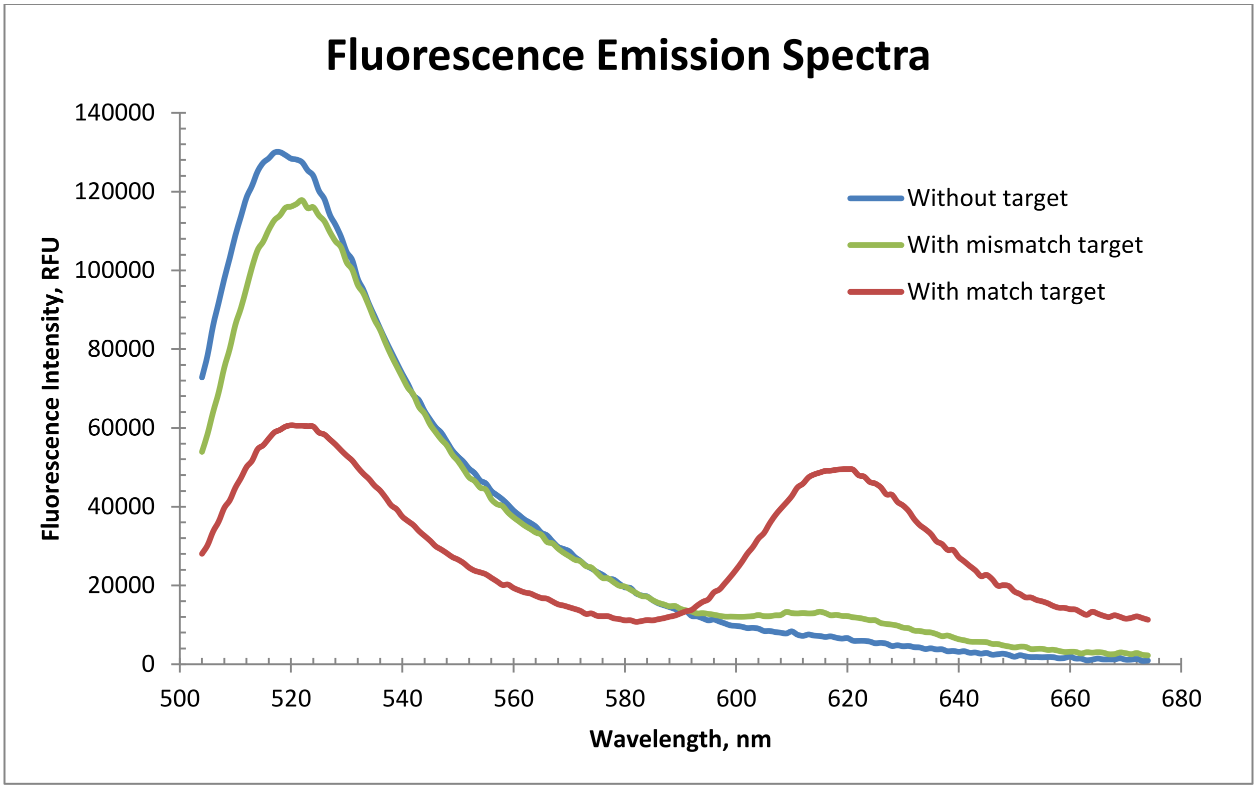

Figure S2. Fluorescence resonance energy transfer demonstrated by fluorescence emission spectra. 200 nM anchor (Oligo 1) and T-allele probe (Oligo 2) were incubated and measured at 37°C without target RNA (Blue line), with mismatch target Oligo 10 (Green line), and with match target Oligo 9 (Red line).
